# Operating room technician trainees teach medical students - an inter-professional peer teaching approach for infection prevention strategies in the operation room

**DOI:** 10.1186/s13756-019-0526-2

**Published:** 2019-05-14

**Authors:** Jan Breckwoldt, Monika Knecht, Ralph Massée, Barbara Flach, Caroline Hofmann-Huber, Sylvia Kaap-Fröhlich, Claudia M. Witt, Ruth Aeberhard, Hugo Sax

**Affiliations:** 1 Faculty of Medicine, Office of the Dean, University of Zurich, Pestalozzistr. 3-5, CH-8091 Zurich, Switzerland; 2 Careum Training Centre, Gloriastrasse 16, CH-8006 Zurich, Switzerland; 3 Department for Education Development, Careum, Pestalozzistr. 3, CH-8006 Zurich, Switzerland; 4 Institute for Complementary and Integrative Medicine, University Hospital Zurich, Raemistrasse 100, CH-8091 Zurich, Switzerland; 5 Department of Infectious Diseases and Hospital Epidemiology, University Hospital Zurich, Raemistrasse 100, CH-8091 Zurich, Switzerland

**Keywords:** Interprofessional education, Operation room technicians, Medical students, Infection prevention, Simulated operating room, Simulation training

## Abstract

**Background:**

Education is a cornerstone strategy to prevent health-associated infections. Trainings benefit from being interactive, simulation-based, team-orientated, and early in professional socialization. We conceived an innovative inter-professional peer-teaching module with operating room technician trainees (ORTT) teaching infection prevention behavior in the operating room (OR) to medical students (MDS).

**Methods:**

ORTT delivered a 2-h teaching module to small groups of MDS in a simulated OR setting with 4 posts*: ‘entering OR’; ‘surgical hand disinfection’; ‘dressing up for surgery and preparing a surgical field’, ‘debriefing’.* MDS and ORTT evaluated module features and teaching quality through 2 specific questionnaires. Structured field notes by education specialist observers were analyzed thematically.

**Results:**

On Likert scales from − 2 to + 2, mean overall satisfaction was + 1.91 (±0.3) for MDS and + 1.66 (±0.6 SD) for ORTT while teaching quality was rated + 1.89 (±0.3) by MDS and self-rated with + 1.34 (±0.5) by ORTT. Students and observers highlighted that the training fostered mutual understanding and provided insight into the corresponding profession.

**Conclusions:**

Undergraduate inter-professional teaching among ORTT and MDS in infection prevention and control proved feasible with high educational quality. Inducing early mutual understanding between professional groups might improve professional collaboration and patient safety.

**Electronic supplementary material:**

The online version of this article (10.1186/s13756-019-0526-2) contains supplementary material, which is available to authorized users.

## Background

An interdisciplinary expert group identified “education & training” as one out of three key strategies with high quality evidence for infection prevention and control (IPC) [1]. In this review it was highlighted that the trainings should be team- and task-orientated, should include hands-on and simulation-based formats, and should rather draw on reflection of individual experience [2] than on “traditional approaches based on logic and reasoning” [3]. While the evidence of education & training was rated high, the ease of implementation was judged to be lower due to financial constraints and lack of teaching experience. The group called for “multimodal and multidisciplinary strategies involving health care workers at all levels”. For the topic of hand hygiene this call has readily been addressed in undergraduate medical education [4–6]. However, to our knowledge teaching of more complex situations to apply IPC standards has not been reported so far. We sought to approach this gap by creating a specific learning module for correct IPC behavior in operating rooms (OR’s).

In complex teams divergent attitudes of professional groups towards IPC have been shown [7–11]. To a certain extent this may be related to stereotypes which usually develop early in professional careers [12, 13]. To counteract such development we introduced the learning module early in the pre-graduate curriculum of medical students (MDS) and OR technician trainees (ORTT). In addition to general teaching and learning principles we drew on three specific well-founded educational strategies: (a) establish a meaningful overarching learning objective [14, 15], in our case: the common goal of patient safety, (b) use an inter-professional setting to contribute to early professional identity formation [16–18] and (c) use near-peer teaching to strengthen the self-confidence of teaching peers in their field of expertise [19–21]. Taken together, we connected patient safety to inter-professional collaboration as it is crucial in OR settings. Specifically, ORTT would teach IPC behavior in the OR to MDS. In this paper we report the concept of the module as well as its quantitative and qualitative evaluation.

## Material and methods

### Design of the inter-professional training module

The inter-professional training module for infection prevention behavior in the OR was planned by a group of specialists from surgical nursing, infection prevention and control, medical education, and inter-professional learning (MK, CHH, JB, HS, RM, BF, SKF). We additionally included recommendations by final-year MDS regarding the skills they had found critical during their clinical clerkship year.

An appropriate curricular time point for ORTT was determined within the second year of training, after 8 months of practical workplace training in ORs - having already gained well-founded competencies in infection prevention behavior. For MDS we embedded the module in their practical surgery course during the first year of clinical studies preparing for later clerkships. For both student groups the module was part of the mandatory curriculum. The planning group agreed on joint learning objectives in the field of inter-professional exchange, including patient safety as an overarching theme, as well as on different content specific learning objectives for each student group. The learning objectives were based on the knowledge and skills acquired during previous trainings, recommendations from the literature [22], and on the “WHO guidelines on hand hygiene in health care” [23]. Specific learning objectives are shown in Table 1.

**Table 1:** Learning outcomes for OR technician trainees, medical students and both student groups

Learning objectives	Psychometric dimension^a^
Overarching (general) learning objectives:	
Infection prevention and control in the operating room	
- Contributes to patient safety	
- Is promoted by inter-professional collaboration	
Specific learning objectives	
At the end of the teaching/learning session ...	
students from both professional groups are able to …	
mutually share the perspective of the complementary pro-fessional group, exchange between the two professional study programs	Cognitive, affective
understand their roles as team players within a culture of patient safety in the OR	Affective, cognitive
describe how they could contribute to a culture of safety even in positions of lower hierarchy (including “speak-up” strategies)	Cognitive, affective
operating room technician trainees (ORTT) are able to …	
pass on well-founded knowledge and skills for antiseptic behavior in the OR to medical students (including consolidation of own competencies)	Cognitive, psycho-motor, affective
be aware of one’s own role as an expert in antiseptic behavior in the OR	Cognitive, affective
reflect their teaching performance based on the experience they gathered within the module	Cognitive, affective
medical students are able to …	
demonstrate effective surgical hand disinfection	Psychomotor
demonstrate appropriate behavior in the OR setting (assignment of tasks, positioning in the room etc.)	Cognitive, psycho-motor, affective
explain principles to prevent germ transmission in the OR	Cognitive
explain and in parts demonstrate the preparation of a surgical field prior to a simple intervention	Cognitive, psycho-motor

^a^simplified Bloom’s taxnonomy (cognitive, psychomotor, and affective objectives) [24]

The teaching module was structured as a sequence of four learning posts mirroring the workflow of a surgical team and took place in a full-scale OR simulator with routine OR consumables. The module featured the following four posts: (1) OR entry procedure including changing of clothes, (2) surgical hand disinfection exercise for all students at the scrub area, (3) dressing up for surgery jointly demonstrated by one ORTT and one MDS, skin disinfection of a simulated surgical field, and application of surgical drapes on a manikin, and (4) re-changing clothes, followed by a debriefing regarding the content of the module, including safety culture and “speak-up” techniques. The modules 1–3 were led by ORTT, module 4 was facilitated by an educational supervisor.

In May 2017, the module took place with 32 ORTT (entire 2nd-year cohort) and 46 MDS (one sixth of the 1st-clinical-year cohort). Eight training groups consisted of four ORTT and five to six MDS each. The ORTT in each group delivered a complete teaching sequence to the group’s MDS. The teaching groups started their way through the four-post parcours in 30-min intervals to optimally use the capacity of the simulation environment.

Prior to their teaching task ORTT were given one half-day for preparation. They familiarized with the theoretical and practical learning content MDS had already dealt with during their studies in microbiology and IPC, then established learning objectives for the MDS, and subsequently practiced teaching sessions. ORTT were familiar to problem-based learning from their own curriculum and were therefore used to set up learning objectives.

### Data collection and analysis

Directly after the course all students filled out a structured 13-item questionnaire to rate various aspects of the training module using Likert scales from − 2 (very poor) to + 2 (excellent) with the opportunity to add free-text comments (Additional file 1). Using a second questionnaire MDS rated the ORTTs’ teaching quality while ORTT self-rated their own teaching. This second questionnaire covered 10 criteria which were all based on empirical evidence from educational science [25–28]. Each item was specified by an example of teaching behavior (Additional file 2). Quantitative data were analyzed by descriptive statistics, comparison of the teaching quality rating by t-test.

To add an external perspective, each student group was observed by an education specialist over the entire sequence of learning posts and the debriefing sessions (MK, RM, BF, SKF, JB). These observations were documented through written notes and were analyzed by inductive thematic analysis [29]. Single comments were assigned to themes and sub-themes and classified as positive, neutral, and negative.

### Ethical consideration

The Ethical Committee of the Canton of Zurich declared no objections to the study (BASEC-2017-00598).

## Results

All 32 ORTT and 46 MDS completed the module. Among the ORTT, 28 were female (87.5%) and the mean age was 24.1 years (SD, 4.4); among the MDS, 27 were female (59%) and the mean age was 22.8 years (SD, 1.9). Previous experience in a real OR setting was 32 weeks for the ORTT, and below 1 week for the MDS. All ORTT groups delivered their teaching within the given time frame. Each MDS had sufficient opportunities to practice entering of the OR area, surgical hand disinfection, and behavior in the OR. One MDS per group could dress up for surgery and - together with an ORTT - jointly perform the preparation of a surgical field.

### Quantitative analysis of the two questionnaires

**Fig. 1:**
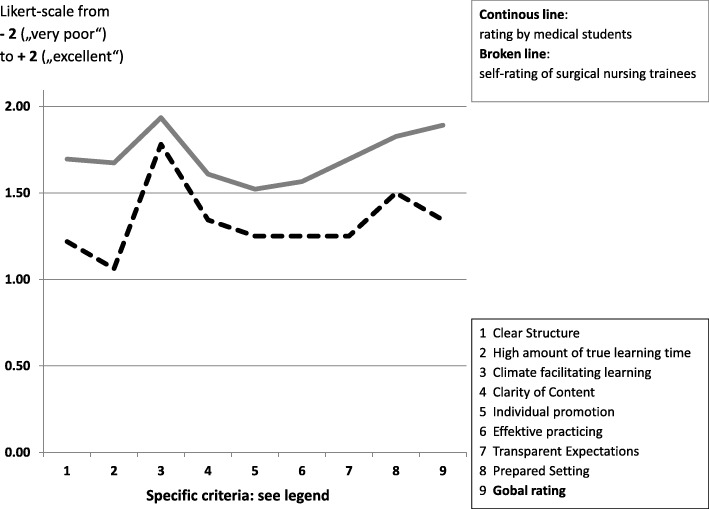
Correlation between self-rating of teaching quality by OR technician trainees and rating by medical students. 8 of 10 categories could directly be compared: mean difference was 0.55 Likert scale points, *p* < .0001 (t-test)

Detailed results from the questionnaire evaluating the whole teaching module are shown in Table 2. MDS rated their learning experience highly positive (with 1.48 being the lowest of all criteria). ORTT rated most items only slightly lower than the MDS with the differences ranging from zero to 0.25 points. Results from the questionnaire for the quality of teaching by ORTT are shown in Table 3. MDS gave very high ratings and ORTT self-rating was also high, however, significantly lower than the ratings by MDS (mean difference: 0.55 points; *p* < .0001). A graphic comparison between both ratings is shown in Fig. 1.

**Table 2:** Evaluation of the inter-professional teaching module: rating by OR technician trainees (ORTT) and by medical students (MDS). Rating on Likert-Scales from −2 (very poor) to + 2 (excellent)

Student group	ORTT ^**a)**^		MDS ^**b)**^	
Number of responses (response rate)	*n* = 32 (100%)		*n* = 46 (100%)	
	mean	SD	mean	SD
Content of the teaching module				
This inter-professional event was a positive learning experience	1.66	0.6	1.91	0.3
This module promoted mutual respect and understanding	1.81	0.4	1.83	0.4
This teaching module prepared well for the future work in an operating room (e.g. during clerkship, or for surgical nursing)	1.22	0.7	1.35	0.7
This teaching module could replace a respective module in a real-life operating room (to train antiseptic behavior in the OR)	-^c)^		0.65	1.0
This workshop provided an insight into the way of thinking and the perspective of the corresponding profession	1.41	0.7	1.59	0.6
This module is helpful to promote inter-professional communication / exchange	1.59	0.6	1.67	0.6
Should your curriculum have more overlap with other professions	1.59	0.6	1.65	0.5

^a^*ORTT* operating room technician trainee

^b^*MDS* medical students

^c^Not relevant for ORTT

**Table 3:** Teaching quality by OR technician trainees (ORTT), rated by medical students (MDS) and self-rated by ORTT. Rating on Likert-Scales from −2 (very poor) to + 2 (excellent)

Evaluation of teaching quality ^**a**)^	Self-rating by ORTT ^**b**^		Rating by MDS ^**c**^	
Clear structure	1.22	0.4	1.70	0.6
High amount of true learning time	1.06	0.7	1.67	0.6
Climate facilitating learning	1.78	0.5	1.93	0.3
Clarity of content	1.34	0.6	1.61	0.7
Meaningful communication	**-** ^**d)**^		1.76	0.5
Variation of methods	**-** ^**e)**^		1.48	0.6
Individual promotion	1.25	0.6	1.52	0.7
Effective practicing	1.25	0.5	1.57	0.8
Transparent expectations	1.25	0.8	1.70	0.6
Prepared setting	1.50	0.7	1.83	0.4
Overall rating	1.34	0.5	1.89	0.3

^a^Ten empirically based criteria for good teaching based on [25–28]

^b^*ORTT* operating room technician trainee

^c^*MDS* medical students

^d^item not provided for ORTT as it was not thought to be suitable for objective self-rating

^e^item not provided to ORTT, because much of the teaching format was predefined and could not be influenced by ORT trainees

### Analysis of questionnaire free text

MDS made free text comments in 18 (of the 46) questionnaires: eight of them were positive, one was negative, and nine were neutral providing recommendations for further improvement. A quotation underlining the generally positive view was: “…*could well be more of this kind of courses*” (8 M6).

ORTT made 12 free text comments (in 32 questionnaires) of which two were positive, two were mixed, three negative, and five neutral suggestions for further development. The ORTT free text comments were more self-critical in respect to the delivery of the module (“…*MDS should be able to train all practical skills*”; 2O1) and to the time devoted to posts 1 and 3.

### Qualitative analysis of observer field notes

The educational observers reported that all ORTT teams managed their teaching task well and showed only minor inconsistencies in regard to content. However, the performance of teams differed ranging from placidity to excitement. Nonetheless, all MDS were observed to be highly attentive over the whole course of the module. All debriefing sessions stimulated an intensive and lively discussion on team collaboration and inter-professional exchange.

Field notes taken by observers during the debriefing sessions were divided between MDS and ORTT to capture the different perspectives of the student groups. Thematic analysis lead to five themes from MDS comments (“*curricular design*”, “*organization of course*”, “*inter-professional setting*”, “*relevance for practice*”, “*delivery of teaching*”) and five themes from ORTT comments (“*curricular design*”, “*organization of course*”, “*inter-professional setting*”, “*delivery of teaching*”, “*reflection on teaching*”). Related sub-themes showed partial overlap in respect to the main themes. Detailed findings including representative comments are shown in Table 4.

**Table 4:** Themes and sub-themes derived from observer filed notes of debriefing sessions, including representative quotes

Themes	n^a^	Sub-themes (pos./ neutr./ neg.)^b^	Representative quotes
MDS			
Curricular design (of MDS)	8	general (4 / 0 / 0)	“… really great to have this module” (DM202)
		teaching content (3 / 0 / 1)	
		practical involvement (1 / 0 / 0)	
Organization of course	2	course preparation (2 / 0 / 0)	“… excellent preparation of the module” (DM104)
Inter-professional setting	6	interprofess. Exchange (3 / 0 / 0)	“[it was] especially [positive] that students [ORTT] delivered it [the course]” (DM701)
		teaching content (2 / 0 / 0)	
		delivery by students (1 / 0 / 0)	
Relevance for practice	5	teaching content (5 / 0 / 0)	“this [learning content] is also important in daily practice” (DM101)
Delivery of teaching	17	practical involvement (5 / 0 / 0)	“There was always someone to provide constructive feedback” (DM205) “It was important that ORTTs had been strict” (DM105)
		teaching / feedback (4 / 0 / 1)	
		learning climate (4/ 0 / 0)	
		teaching quality (2 / 0 / 0)	
		teaching strategies (2 / 0 / 0)	
sum (MDS)	38	(35 pos./ 0 neutr./ 3 neg.)	
ORTT			
Curricular design (of ORTT)	12	time management (1 / 1 / 5)	“30 min for each post was tough timing” (DO107)
		teaching content (0 / 0 / 2)	
		teaching strategies (2 / 0 / 0)	
		practical instruction (0 / 0 / 1)	
		general (1 / 0 / 0)	
Organization of course	3	course preparation (1 / 0 / 2)	“Preparation time [provided by school] was too short” (DO404)
Inter-professional setting	1	interprofess. Exchange (1 / 0 / 0)	[positive:] “.. integrating different professional fields” (DO401)
Delivery of teaching	3	practical instruction (1 / 0 / 0)	“All were encouraged to try things out” (DO306)
		learning climate (0 / 0 /1)	
		teaching content (1 / 0 / 0)	
Reflection on teaching	11	delivery of teaching (1 / 1 / 3)	“Experience of teaching med students: surprisingly positive” (DO508) “It was difficult to decide what to prepare” (DO706) “I was nervous - for the theory part” (DO307)
		teaching content (0 / 1 / 1)	
		experience to teach (1 / 0 / 0)	
		time management (0 / 0 / 1)	
		teaching strategies (0 / 1 / 0)	
sum (ORTT)	30	(8 pos./ 5 neutr./ 17 neg.)	

^a^number of comments; ^b^ classification: positive / neutral / negative)

Comments made by MDS were almost exclusively positive (35 out of 38) with a focus on the theme “*delivery of teaching*” (17/38). Most important sub-themes were “*practical involvement*”, “*teaching/feedback*”, and “*learning climate*”. MDS also highlighted the relevance for clinical practice and stated that they had reached a new understanding of ORTT being “*experts of OR hygiene*” (DM501).

Comments from ORTT put more weight on critical aspects resulting in 17 negative comments out of 30 (see Table 4). These comments mainly covered the themes “*curricular design*” and “*reflection on teaching*”. ORTT felt that time for teaching was limited, and they critically reflected on their own teaching in terms of delivery, content, and strategies. In addition, ORTT recommended to devote more time to post 3 (“dressing up for surgery and preparing a surgical field”) to provide individual practice for all MDS. Further critical comments related to the perceived short period the ORT curriculum had assigned for the teaching task. However, ORTT predominantly appreciated the teaching experience (quotation: “*teaching experience was surprisingly positive*” (DO508)). Typical areas mentioned for improvement were “prioritizing content and learning objectives*”*, “managing the time”, and “managing one’s own nervousness”. In the field of inter-professional exchange ORTT stressed to have gained a new understanding of the situation of MDS in the OR.

The culture of dealing with medical errors was specifically addressed by supervisors during debriefing. Within all student groups the observers found that explicit “speak-up “techniques were not known. Notwithstanding, in half of the groups ORTT were able to describe and explain effective ways to address poor adherence to safety standards and medical errors from the perspective of a lower hierarchical position.

Both students groups emphasized that the module fostered mutual respect and provided an excellent opportunity to gain insight into the other profession’s perspective. More opportunities for inter-professional exchange during education were requested. Notably, students from both groups found it a very positive experience having been at a similar stage of training.

## Discussion

Promoting adherence to infection prevention standards in complex team settings calls for special teaching and learning strategies. In this paper we presented an inter-professional peer-teaching module in early undergraduate training using innovative teaching strategies. The module proved feasible and we found various indicators of high teaching quality. The success may be explained by the combination of meaningful learning objectives, placing the teaching intervention early in lifelong learning and employing an inter-professional near-peer teaching concept. These factors will be discussed in the following.

### Meaningful learning outcomes

First and above all, it was important to establish patient safety as the final overarching learning outcome. Thereby we linked the – rather abstract – theme “infection prevention” to an emotionally engaging collaborative goal of an OR team. This clearly legitimated the module within both curricula and made active participation meaningful for both student groups. The second important learning objective was to make clear that inter-professional collaboration is a key to achieve patient safety in complex team settings. Qualitative and quantitative results of this study strongly support that this goal had been reached. A mutual understanding of the complementary professional groups was clearly stated by both student groups in the questionnaires and during the debriefing sessions. Meaningful exchange may partly be attributed to the educational supervisors who moderated the debriefing sessions and it remains open whether ORTT had been capable to facilitate this session. Nevertheless, both student cohorts intensely discussed strategies to improve patient safety by improving team performance and applying “speak-up” techniques.

While providing a shared perspective on inter-professional collaboration to achieve patient safety the specific learning outcomes for MDS (OR hygiene, surgical hand disinfection and appropriate behavior in the OR) and for ORTT (teaching skills, and becoming aware of one’s expert knowledge) could be placed in a meaningful context.

### Early professional identity formation

As a second factor of success, the module was placed in an inter-professional setting early in lifelong learning. Both student groups were at a comparable stage of their training. This provided a shared perspective for future team collaboration and may be regarded as a very early “practice-based intervention” in the concept of inter-professional education [30, 31]. Students’ comments underline that appropriate curricular time points had been selected for the module. We did not note any complaints of the learning content being too elementary, or that prior knowledge for this module was lacking.

Dealing with errors in inter-professional settings turned out to be a central topic which apparently had not been addressed explicitly in either of the preceding curricula. In consequence, our module provided the opportunity to introduce the concept of “speaking-up” including its inherent difficulties. Thereby once more professional identity formation could be promoted in a direction of inter-professional collaboration to serve patient safety.

### Near-peer teaching to strengthen the self-confidence of ORTT

As a third factor of success we regard the near-peer teaching approach. ORTT made the experience of having successfully mastered the teaching task. It is likely that this contributed to ORTT self-efficacy [32], as some of the ORTT stated, and it is consistent with the findings in other peer teaching settings [33, 34]. MDS rated the teaching quality of ORTT very high, and highly acknowledged the competence of ORT trainees in their field of expertise. ORTT for their part self-rated their teaching lower than the MDS did and reflected critically on their teaching during debriefing. This may indicate an active involvement of ORTT. All eight criteria of teaching quality, which could be compared between the student groups, showed a concordant pattern, which may be taken as an indication for realistic self-assessment. This finding also adds validity to the evaluation tool.

The experience to “pass on well-founded knowledge and skills” was made by all ORTT, although the extent varied. The group size of four gave the opportunity to share the teaching tasks, therefore not being exposed as a single instructor and having the option to step into the back. On the other hand, this could as well have been tempting to hide behind the rest of the group and avoid deeper involvement. Therefore, it may be discussed to reduce the number of ORTT when further developing the module (e.g. to two ORTT).

Whether ORTT had become more aware of their role as experts for infection prevention in the OR, may also only be estimated from the observations made in the debriefing sessions. The dedicated discussions on the significance of patient safety may be taken as an indicator for increased awareness in this field. Still, it can neither exclusively be attributed to the teaching module, nor can it be concluded that a sustainable effect for further practice was evoked. Further research should explore this question in more depth.

### Teaching setting

The infrastructure was ideal for a standardized teaching setting with a high degree of realism and the important opportunity to provide timely and specific feedback [35]. In addition, near-peer-teaching may lower costs by reducing the need for regular faculty [21].

However, MDS stated that the module could only partially replace a respective learning experience in real-life ORs. Further studies should therefore explore whether elements of our module could be transferred to real ORs. The training post “behavior in the OR” may in future be split into two successive stations to give more time for practice to each MDS.

### Limitations

This study holds limitations, starting with its non-controlled, mainly descriptive nature. Outcomes are restricted to qualitative observations and evaluation at the “reaction” level (level 1 according to Kirkpatrick [36]). We are unable to provide evidence that actual learning of either student group had occurred. Subsequent research needs to address these issues using randomized controlled settings and including the assessment of students and trainees. Further limitations include the mono-centered setting and social desirability bias (which cannot be ruled out even though questionnaires were filled out anonymously). Taken together, justification for generalization is low. Nonetheless, we argue that this project offers a number of important insights into teaching principles which worked well for the subject of complex team situations in the context of infection prevention.

## Conclusions

We introduced an inter-professional teaching format during ORTT and undergraduate medical education to promote infection prevention in the context of OR teams. The teaching format was feasible, learning objectives were sufficiently covered, and the educational quality was rated high. On an inter-professional level the module promoted early mutual understanding of professional groups. In summary, this combination of well-founded educational strategies appears to ideally meet the proposed educational requirements to improve infection prevention and control.

## Additional files

Additional file 1: Questionnaire “Evaluation of the teaching module”. (DOCX 271 kb) (DOCX 271 kb)

Additional file 2: Feedback questionnaires for ORT trainees and for medical students. (DOCX 258 kb) (DOCX 258 kb)

## Abbreviations

IPC: Infection prevention and control
MDS: Medical students
OR: Operating room
ORTT: Operating room technicians
WHO: World Health Organization
