# Supplementary figures and images for: Operating room technician trainees teach medical students - an inter-professional peer teaching approach for infection prevention strategies in the operation room
Source: Antimicrob Resist Infect Control. 2019 May 14;8:75. doi: 10.1186/s13756-019-0526-2 (PMC6518629; doi:10.1186/s13756-019-0526-2)

**Supplementary File 1**: Questionnaire “Evaluation of the teaching module”


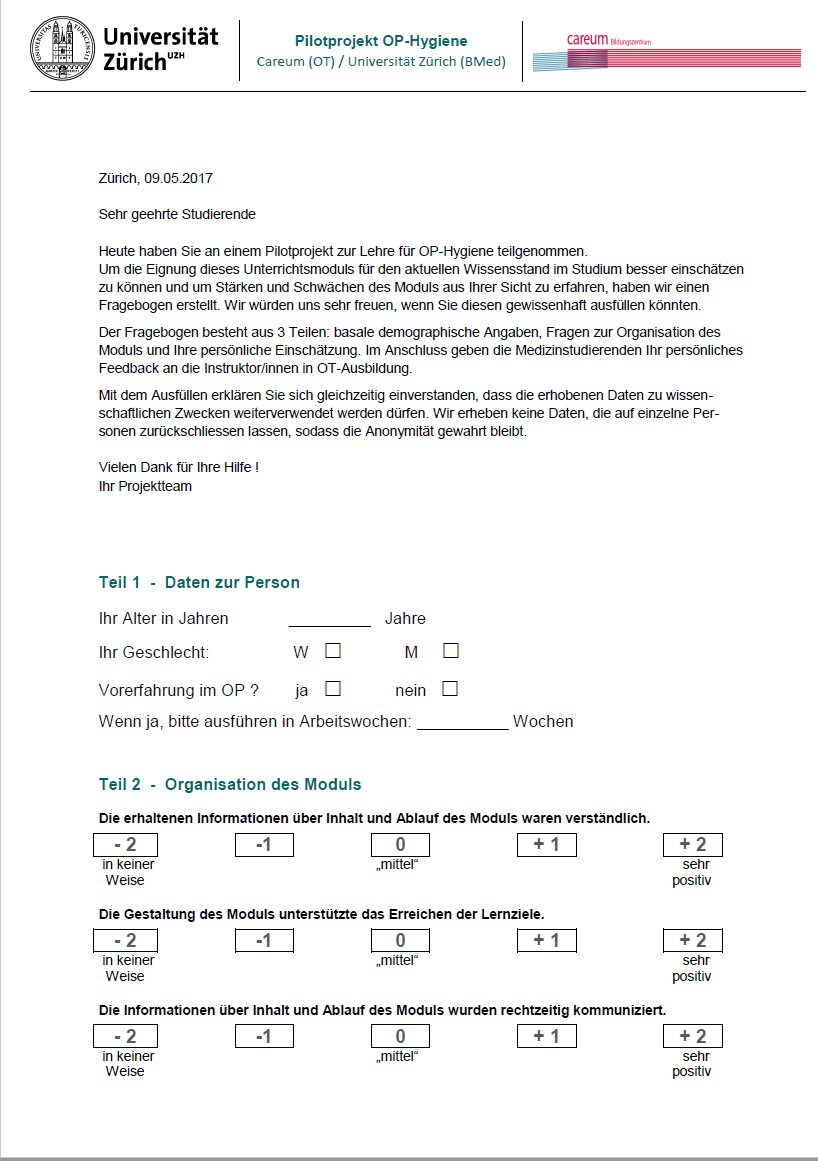


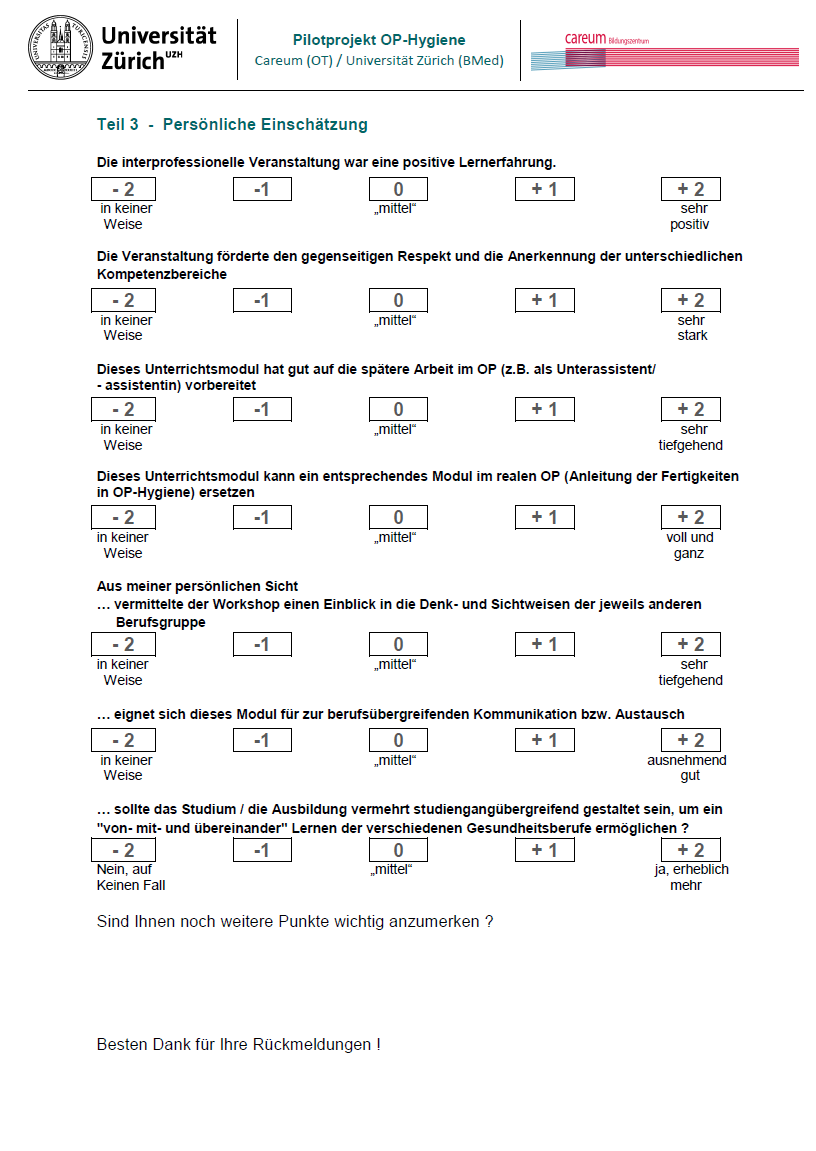

Supplement: Supplementary file 1 — Questionnaire “Evaluation of the teaching module”. (DOCX 271 kb) (DOCX 271 kb) [file 13756_2019_526_MOESM1_ESM.docx]
