# Supplementary material for: Operating room technician trainees teach medical students - an inter-professional peer teaching approach for infection prevention strategies in the operation room
Source: Antimicrob Resist Infect Control. 2019 May 14;8:75. doi: 10.1186/s13756-019-0526-2 (PMC6518629; doi:10.1186/s13756-019-0526-2)

**Supplementary File 2**

Questionnaires to rate for OR technician trainees’ teaching quality, Version for medical students (OR technician trainee version: without item 5 (“meaningful communication”), and item 6 (“variation of methods”).


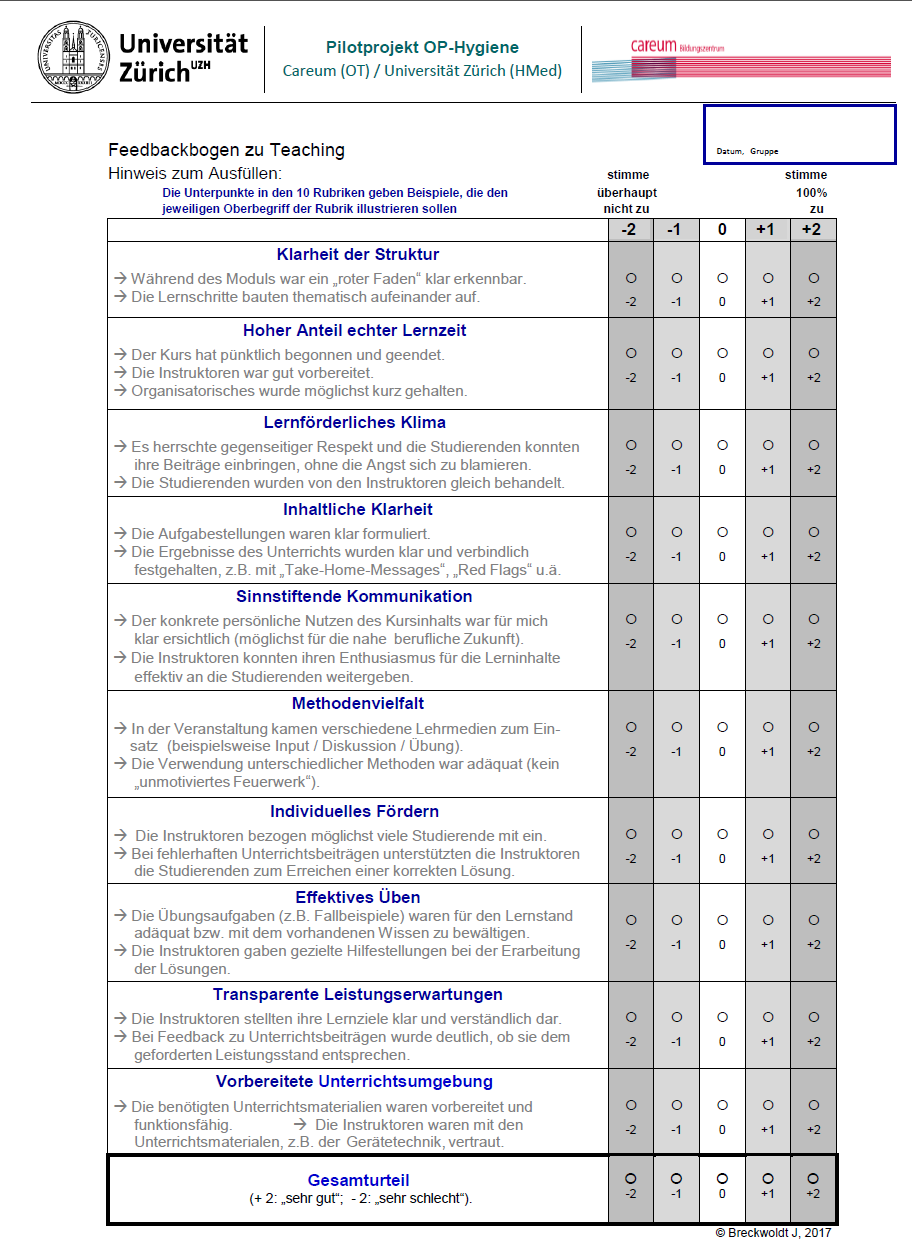

Supplement: Supplementary file 2 — Feedback questionnaires for ORT trainees and for medical students. (DOCX 258 kb) (DOCX 258 kb) [file 13756_2019_526_MOESM2_ESM.docx]
